# TCTP from *Loxosceles Intermedia* (Brown Spider) Venom Contributes to the Allergic and Inflammatory Response of Cutaneous Loxoscelism

**DOI:** 10.3390/cells8121489

**Published:** 2019-11-22

**Authors:** Marianna Boia-Ferreira, Kamila G. Moreno, Alana B. C. Basílio, Lucas P. da Silva, Larissa Vuitika, Bruna Soley, Ana Carolina M. Wille, Lucélia Donatti, Katia C. Barbaro, Olga M. Chaim, Luiza Helena Gremski, Silvio S. Veiga, Andrea Senff-Ribeiro

**Affiliations:** 1 Department of Cell Biology, Federal University of Paraná, Curitiba 81531-980, PR, Brazil; marianna.boia@gmail.com (M.B.-F.); kamilamoreno7@gmail.com (K.G.M.); alana_abcb@hotmail.com (A.B.C.B.); spedrosa.lucas@gmail.com (L.P.d.S.); larissavuitika2@gmail.com (L.V.); donatti@ufpr.br (L.D.); olgachaim@ufpr.br or ochaim@ucsd.edu (O.M.C.); luiza_hg@yahoo.com.br (L.H.G.); veigass@ufpr.br (S.S.V.); 2 Department of Pharmacology, Federal University of Paraná, Curitiba 81531-980, PR, Brazil; brunasoley@gmail.com; 3 Department of Structural and Molecular Biology, State University of Ponta Grossa, Ponta Grossa 84030-900, PR, Brazil; anacarolina.wille@yahoo.com.br; 4 Laboratory of Immunopathology, Butantan Institute, São Paulo 05503-900, SP, Brazil; katiacbarbaro@hotmail.com; 5 Department of Pharmacology, School of Medicine, University of California San Diego, La Jolla, CA 92093, USA

**Keywords:** *Loxosceles*, brown spider, TCTP, venom, toxin, HRF

## Abstract

LiTCTP is a toxin from the Translationally Controlled Tumor Protein (TCTP) family identified in *Loxosceles* brown spider venoms. These proteins are known as histamine-releasing factors (HRF). TCTPs participate in allergic and anaphylactic reactions, which suggest their potential role as therapeutic targets. The histaminergic effect of TCTP is related to its pro-inflammatory functions. An initial characterization of LiTCTP in animal models showed that this toxin can increase the microvascular permeability of skin vessels and induce paw edema in a dose-dependent manner. We evaluated the role of LiTCTP in vitro and in vivo in the inflammatory and allergic aspects that undergo the biological responses observed in Loxoscelism, the clinical condition after an accident with *Loxosceles* spiders. Our results showed LiTCTP recombinant toxin (LiRecTCTP) as an essential synergistic factor for the dermonecrotic toxin actions (LiRecDT1, known as the main toxin in the pathophysiology of Loxoscelism), revealing its contribution to the exacerbated inflammatory response clinically observed in envenomated patients.

## 1. Introduction

LiTCTP is a protein from the Translationally Controlled Tumor Protein (TCTP) family that was found in the *Loxosceles intermedia* brown spider venom, initially in a cDNA library of the *L. intermedia* venom gland and confirmed in the transcriptome analysis of the venom gland [1,2]. Although it represented only 0.4% of the toxin-related transcripts, it was positively identified by immunodetection in the whole venom of different *Loxosceles* species (*L. intermedia*, *L. gaucho*, and *L. laeta*) [3], which indicates its biological conservation and importance. TCTP proteins are known as histamine releasing factors (HRF) and activators of mast cells and basophils triggering the release of histamine [4,5]. Mast cells are intimately involved in allergic and anaphylactic reactions, and an increasing body of evidence involves these cells and their mediators in the pathophysiology of inflammation [6]. *Loxosceles* venoms are responsible for severe skin lesions at the bite site, characterized by intense inflammatory content, which can evolve to necrotic conditions [7,8]. Hypersensitivity or even allergic reactions are also reported as clinical features of Loxoscelism [9,10]. Mast cells were already mentioned as involved in biological responses evoked by *Loxosceles* venom toxins, as inflammatory response was partially reduced in compound 48/80-pretreated animals [11]. Previous study of LiRecTCTP, the recombinant isoform of LiTCTP, showed this toxin increases microvascular permeability of skin vessels, causing a diffuse pattern of dye leakage. Moreover, LiRecTCTP induced paw edema, which reached its maximum after 5 min of inoculation (an early effect compared to dermonecrosis) [2]. TCTP was already described as a putative therapeutical target in asthma and allergy due to its pro-inflammatory extracellular effects [5,12]. Herein, we studied the participation and effects of LiRecTCTP toxin in the biological histaminergic and inflammatory response observed in Loxoscelism. LiRecTCTP was also studied in combination with the well-known LiRecDT1 Brown spider toxin, a recombinant isoform of phospholipase-D (PLD) of *L. intermedia*. LiRecDT1 can cause the majority of whole venom-induced effects in vitro and in vivo. Meanwhile, LiRecTCTP was shown to enhance the biological response to LiRecDT1, pointing to its contribution to the exacerbated inflammatory response observed clinically in the patients. Our findings strengthen the idea of inhibition of LiTCTP and mast cell activation effects as promising therapeutic approach to reduce the inflammatory events responsible for the main symptoms in cutaneous Loxoscelism.

## 2. Materials and Methods

### 2.1. Reagents

The whole venom from *L. intermedia* was obtained by electrostimulation (15 V) of the cephalothorax of spiders, solubilized in PBS, and maintained frozen until use [13]. *L. intermedia* spiders were captured in the wild with the authorization of the Brazilian Governmental Agency “Instituto Chico Mendes de Conservação da Biodiversidade” (Number 29801-1). Ni^2+^-NTA agarose was purchased from Invitrogen (Carlsbad, CA, USA). DMEM media were purchased from Gibco (Carlsbad, CA, USA). The molecular mass markers were acquired from Sigma Aldrich (St. Louis, MO, USA). Evans Blue dye was purchased from Vetec (São Paulo, Brazil). The Compound 48/80, cromolyn sodium salt (cromolyn), promethazine hydrochloride (promethazine), cimetidine hydrochloride (cimetidine), and thioperamide maleate salt (thioperamide) were purchased from Sigma Aldrich. Ketamine and Sedanew^®^ (xylazin 10%) were from Agribands (Campinas, Brazil) and Univet (São Paulo, Brazil), respectively.

### 2.2. Recombinant Protein Expression

The pET-14b cDNA construct [2] was transformed into one-shot *E. coli* BL21(DE3) pLysS competent cells (Invitrogen), plated on LB agar medium containing ampicillin (100 µg/mL) (Sigma Aldrich), and chloramphenicol (34 µg/mL) (Sigma Aldrich). One colony was then incubated in 10 mL of LB broth (with antibiotics) and allowed to grow overnight at 37 °C under orbital agitation. Then, this pre-culture was expanded into 1L of LB broth with antibiotics and allowed to grow at 37 °C until the OD at 550 nm reached 0.5. For the induction of heterologous protein expression, isopropyl-d-thiogalactoside (IPTG, ThermoFisher Scientific, Waltham, MA, USA) was added at a final concentration of 0.1 mM, and induction of the culture was performed for 4 h at 23 °C.

### 2.3. Recombinant Protein Purification

LiRecTCTP was purified by affinity chromatography using Ni^+2^-NTA column (Invitrogen) as described by Sade and colleagues [2], with modifications. Briefly, *Escherichia coli* cells were lysed by thaw–freeze cycles and disrupted by cycles of sonication. The cell lysate was centrifuged (20,000× *g*, 20 min, 4 °C), and the supernatant was incubated with 1 mL Ni^2+^-NTA beads for 1 h at 4 °C. The recombinant protein was washed with wash buffer (50 mM sodium phosphate pH 6.0, 500 mM NaCl, 35 mM imidazole, Sigma Aldrich), eluted with elution buffer (50 mM sodium phosphate pH 8.0, 500 mM NaCl, 350 mM imidazole), and analyzed by 12.5% SDS-PAGE under reducing conditions. Wash buffer pH was important to the astringent condition for protein binding to the column.

### 2.4. Protein Quantification and Gel Electrophoresis

The total protein concentration of different samples and, especially, the purified LiRecTCTP were determined by the Coomassie Blue method (BioRad, Hercules, CA, USA) [14]. For protein quality analysis, 12.5% SDS-PAGE (sodium dodecyl sulfate-polyacrylamide gel electrophoresis) was performed under reducing conditions.

### 2.5. Circular Dichroism Spectroscopy (CD)

Recombinant LiRecTCTP was dialyzed at 4 °C against a phosphate buffer (20 mM NaH_2_PO_4_/Na_2_HPO_4_ and 150 mM NaCl at pH 7.4) to a final concentration of 0.5 mg/mL. The UV-CD spectra were obtained in a Jasco J-810 spectropolarimeter (Jasco Corporation, Tokyo, Japan) using a 1 mm cuvette, as described previously [15]. The final spectrum (0.5 nm intervals) was the average of three measurements, performed at a rate of 50 nm/min, using a response time of 8 s and a bandwidth of 1 nm. The temperature was kept constant at 20 °C. The data units were expressed as molar ellipticity and plotted by GraphPad Prism 6 software. The results of deconvolution analyses and percentages of secondary structure were predicted by K2D3 tool (https://onlinelibrary.wiley.com/doi/abs/10.1002/prot.23188).

### 2.6. Cell Culture

RBL-2H3 cell line was obtained as a courtesy of Professor Maria Celia Jamur (University of São Paulo, School of Medicine, Ribeirão Preto, SP, Brazil) and were grown in DMEM medium supplemented with 10% FCS (Gibco) and antibiotics penicillin (10,000 U/mL) (Sigma Aldrich) and streptomycin (10 mg/mL) (Sigma Aldrich). RBL-2H3 cells are a rat basophilic leukemia cell line used as a mast-like cell model [16]. RBL-2H3 cells grow as a monolayer, were subcultured (using trypsin/EDTA 2 mM, Gibco) and used until the 10th passage. Cell cultures were kept at 37 °C in a humidified atmosphere with 5% CO_2._

### 2.7. MTT Assay

RBL-2H3 cells (5 × 10^4^ cells per well) cells were plated in 96-well plates and then grown in medium containing 10% FCS at 37 °C in a humidified 5% CO_2_ incubator. After 16 h, cells were washed with Tyrode’s Buffer (137 mM NaCl, 2.7 mM KCl, 1mM MgCl_2_, 1.8 mM CaCl_2_, 0.2 mM Na_2_HPO_4_, 5.5 mM Glucose, 10 mM HEPES, and 0.1% BSA), and then incubated with LiRecTCTP (10, 50, and 100 µg/mL), total venom from *L. intermedia* (10, 50, and 100 µg/mL), 48/80 compound (100 µg/mL) (positive control for degranulation), and PBS or the recombinant toxin LiRecDT1H12A (100 µg/mL) (as negative controls). After 2 h, media was removed and replaced by MTT solution (0.5 mg/mL) (Sigma Aldrich). It is important to mention that incubation with toxins did not cause any detachment of cells from the plates. Cells were again incubated for 3 h at 37 °C. The MTT solution was removed, and formed formazan crystals of each sample were solubilized with DMSO (100 µL) (Sigma Aldrich). The dehydrogenases activity for cell viability assessment was quantified spectrometrically in 550 nm. MTT assay was performed in pentaplicate, and the results are shown as mean ± s.d. of three independent experiments.

### 2.8. In vitro Mast Cell Degranulation Induced by LiRecTCTP

The release of granular beta-hexosaminidase enzyme was measured in the supernatants obtained from RBL-2H3 rat cell line exposed to the recombinant toxins. For this, 5 × 10^4^ cells were plated in medium with 10% FCS. After 16 h, cells were washed, and the medium was replaced by Tyrode’s buffer containing LiRecTCTP (10, 50, and 100 µg/mL) with or without cromolyn (10 µM), total venom of *L. intermedia* (10, 50, and 100 µg/mL), 48/80 compound (100 μg/mL) (positive control), and PBS or the recombinant toxin LiRecDT1H12A (100 µg/mL) (as negative controls) for 2 h at 37 °C in a humidified 5% CO_2_ incubator. From each experimental sample to be quantified, five aliquots (10 µL) of the supernatants were taken as pentaplicates to another microwell plate. RBL-2H3 cells incubated only with the Tyrode’s (TGB) buffer were lysed with 0.5% Triton X-100 (200 µL) (Sigma Aldrich) to evaluate the total enzyme content as 100% reference. To all replicates, 90 µL of the substrate solution containing 1.3 mg/mL of p-nitrophenyl-*N*-acethyl-b-d-glucosamine (Sigma Aldrich) in 0.1 M citrate solution (pH 4.5) were added, and plates incubated for 30 min at 37 °C. Reactions were stopped by addition of 100 µL of 0.2 M glycine solution (pH 10.7), and OD at 405 nm was determined. The extent of secretion was expressed as the percentage of the total beta-hexosaminidase activity in the wells discounted of the values obtained in the supernatant of unstimulated cells [17]. Beta-hexosaminidase activity results are shown as mean ± s.d. of three independent experiments.

### 2.9. Scanning Electronic Microscopy (SEM)

Previously plated on glass coverslips, RBL-2H3 cells were exposed to PBS (as negative control) and LiRecTCTP (100 and 200 µg/mL) in Tyrode’s Buffer (TGB) for 2 h at 37 °C. After, cells were washed with TGB and fixed with Karnovsky (2% formaldehyde, 2.5% glutaraldehyde in 0.1 M of sodium cacodylate buffer pH 7.2 at 4 °C) [18]. Then, fixed cells were dehydrated, and critical-point drying was performed using a Balzers CPD-010 (Balzers Instruments, Balzers, Liechtenstein) with carbonic gas. Metallization in gold was performed using a Balzers SCD-030 (Balzers Instruments). The samples were observed and photographed with a JEOL-JSM 6360 LV scanning electron microscope (JEOL Ltd., Tokyo, Japan) at the Electron Microscopy Center, Federal University of Paraná (Curitiba, PR, Brazil).

### 2.10. Calcium Influx Assay

Calcium influx was measured as previously described [19,20]. Briefly, cultured RBL-2H3 cells were removed and washed with PBS. After, cells were loaded with Fluo-4 AM (10 μM) (ThermoFisher) in buffer with Pluronic F-127 (0.01%) for 30 min at 37 °C. Subsequently, cells were washed twice with Tyrode’s (TGB) buffer without calcium and equilibrated for 30 min at room temperature. Then, 5 × 10^5^ cells/well were incubated in 96 wells black plates with PBS (negative control), LiRecTCTP (50 and 100 μg/mL), and LiRecTCTP (100 μg/mL) combined with cromolyn (10 µM) for 5, 15, 30, 60, and 90 min. The resulting fluorescence was quantified in Tecan Infinite M200 spectrofluorometer (Tecan, Männedorf, Switzerland) using an excitation wavelength of 485 nm and measuring emission at 535 nm. Calcium influx assay was performed in triplicate, and the results are shown as mean ± s.d. of three independent experiments.

### 2.11. Quantitative PCR

Total RNA was extracted from cells using TRIzol Reagent (Life Technologies, Carlsbad, CA, USA) according to the manufacturer’s instructions. RNA was converted in cDNA using High-Capacity RNA-to-cDNA^TM^ kit (Applied Biosystems, Foster City, CA, USA). The cDNA concentrations were measured with a NanoVue Plus spectrophotometer (GE Healthcare, Chicago, IL, USA) and 50 ng cDNA was used. Real-time quantitative PCR was performed using the Power SYBR Green PCR Master Mix in Step One Plus Real-Time PCR System (Applied Biosystems). Primers for IL-3 (sense 5′-ACAATGGTTCTTGCCAGCTCTAC-3′ antisense 5′-AGGAGCGGGAGCAGCAT-3′), IL-4 (sense 5′-CAGGGTGCTTCGCAAATTTTAC-3′ anti-sense 5′-ACCGAGAACCCCAGACTTGTT-3′), IL-13 (sense 5′-GCTCTCGCTTCGCTTGGTGGTC-3′ anti-sense 5′CATCCGAGGCCTTTTGGTTACAG-3′), and GAPDH (sense 5′-TTCACCACCATGGAGAAGGC-3′ antisense 5′- GGCATGGACTGTGGTCATGA-3′) were based on validated sequences from Primer Bank [21]. GAPDH mRNA was used to normalize data, with fold change calculated by the comparative Ct method (ΔΔCT method), as previously described [22]. Results are shown as mean ± s.d. of three independent experiments.

### 2.12. Animals

Adult Swiss mice (25–30 g) from the Central Animal House of the Federal University of Paraná and adult rabbits (3 kg) from CPPI (Centro de Pesquisa e Produção de Imunobiológicos, Piraquara, Brasil) were used for in vivo experiments with the whole venom of *Loxosceles intermedia* and/or the recombinant toxins (LiRecTCTP, LiRecDT1, and GFP). All procedures involving animals were carried out in accordance with “Brazilian Federal Laws,” following the Institutional Ethics Committee for Animal Studies Guidelines from Federal University of Paraná, which approved the project methodology concerning animal studies (Approval Certificates Numbers 730 and 1183).

### 2.13. Pharmacological Treatments (In Vivo)

The following pretreatments were applied into mice in order to investigate LiRecTCTP effects on edema formation and vascular permeability in vivo: cromolyn (mast cell degranulation inhibitor), 30 mg/kg, administered i.p. in three consecutive days before exposure to LiRecTCTP; promethazine (histamine type 1 receptor (H1R) antagonist), 5 mg/kg, administered i.v. 30 min before exposure to LiRecTCTP; cimetidine (histamine type 2 receptor (H2R) antagonist), 15 mg/kg, administered i.p. 2 h before exposure to LiRecTCTP; thioperamide (histamine type 3 and 4 receptor (H3/H4R) antagonist), 20 mg/kg, administered i.p. 30 min before exposure to LiRecTCTP [23]. Sterile PBS was used as a negative control for pharmacological treatments and LiRecTCTP injections.

### 2.14. Effects on Vascular Permeability

Changes in vascular permeability were assessed by visualizing extravasation of Evans Blue dye into the extravascular compartment of the skin [2,24,25]. Briefly, groups of five mice (per condition) were treated with the different types of inhibitors (as previously described in Pharmacological Treatments item). A dilution of the dye in PBS solution (30 mg/kg) was injected intravenously (100 µL) prior to intradermal injection of LiRecTCTP (10 µg) or PBS (negative control) (50 µL). After 60 min, animals were euthanized (intraperitoneal injection of ketamine 30 mg/kg and xylazin 5 mg/kg), and dorsal skin was removed for visualization of dye leakage and photographed. The patches of skin were excised and incubated in 2 mL of formamide at room temperature for five days, after which the absorbance of the resulting supernatant was measured at 595 nm. Results are of one representative experiment from three independent biological replicates (data shown in Figure 4). Alternatively, groups of five mice (per condition) were injected intravenously (100 µL) with a dilution of Evans Blue in PBS solution (30 mg/kg) prior to intradermal injection of LiRecTCTP (5 µg and 10 µg), LiRecDT1 (1 µg), recombinant GFP (10 µg) (negative control), and the same volume of PBS (50 µL) (negative control). After 60 min, animals were euthanized (as described above), and dorsal skin was removed for visualization of dye leakage and photographed (data shown in Figure 5).

### 2.15. Paw Edema-Forming Activity

Paw edema development was measured at different time intervals as previously performed [2,26]. Briefly, groups of five mice (per condition) were treated with different types of inhibitors (as previously described in Pharmacological Treatments item) and were injected subcutaneously into the right hind paw with LiRecTCTP (10 µg dissolved in sterile PBS). Negative control mice were injected with the same volume of PBS (30 µL). Edema was evaluated by examining changes in paw thickness using a calibrated digital micrometer (Digimess, São Paulo, SP, Brazil) at the following time points: immediately after subcutaneous injection (t zero), 5, 10, 20, 30, 60, 120, 240, 360, and 720 min after injection. Results are shown as mean ± s.e.m of one representative experiment from three independent biological replicates.

### 2.16. Dermonecrosis In Vivo

For assessment of dermonecrotic effects, 10 and 20 μg of LiRecTCTP, 1 µg of LiRecDT1 (wild type dermonecrotic toxin), and 20 µg of GFP (a recombinant protein without relevant biological activity) [2,27] were injected subcutaneously into a shaved area of the rabbit dorsal skin. Animals were observed over the course of dermonecrotic lesion progression. Animal skin was photographed immediately after injection and after 24 h of toxins application. The same rabbit received all the 7 samples (divided in the both dorsal sides of the animal) (PBS, GFP, LiRecDT1, LiRecTCTP 10 μg, LiRecTCTP 20 μg, LiRecDT1+LiRecTCTP 10 μg, and LiRecDT1+ LiRecTCTP 20 μg). Experiments were initially performed in two animals and then repeated using four animals. Images show a photograph from the skin of a representative rabbit. Animals were euthanized using intramuscular injection of ketamine (240 mg/kg) and xylazin (27 mg/kg). After euthanasia of animals, skin samples were harvested for histopathological analysis and myeloperoxidase (MPO) activity assay.

### 2.17. Histological Methods for Light Microscopy

Rabbit skin pieces from animals, which were previously subcutaneously inoculated with recombinant toxins, were collected. The tissue samples were fixed in “ALFAC” (ethanol 85%, formaldehyde 10%, and glacial acetic acid 5%) for 16 h at room temperature. After fixation, samples were dehydrated in a graded series of ethanol before paraffin embedding (for 2 h at 58 °C). Then, thin tissue sections (4 μm) were processed and stained with hematoxylin and eosin (H & E). Histological sections were analyzed in Image J analysis software (v.1.x) to quantify edema formation by the measurement of the area between the epidermis and adipose tissue, histological section area observed after GFP protein inoculation was considered the control for comparisons.

### 2.18. Myeloperoxidase (MPO) Activity Assay

The activity of tissue myeloperoxidase (MPO) in rabbit skin was evaluated 24 h after subcutaneous injection of toxins in the animals as previously described [27]. Briefly, a 6 mm skin tissue punch (biopsy) was placed in 0.75 mL of 80 mM sodium-phosphate buffer, pH 5.4, containing 0.5% hexadecyltrimethylammonium bromide (HTAB) (Sigma Aldrich), and then homogenized (45 s at 0 °C) in a motor-driven homogenizer. The homogenates were decanted into a microfuge tubes, and the vessel was washed with 0.75 mL of HTAB-buffer. The wash was added to tube, and the 1.5 mL sample was centrifuged at 12,000× *g* at 4 °C for 15 min. Samples in triplicate (30 μL) of the resulting supernatant were added into 96-well microtiter plates. For the assay, 200 μL of a mixture containing 100 μL of 80 mM PBS (pH 5.4), 85 μL of 0.22 M PBS (pH 5.4), and 30 μL of 0.017% hydrogen peroxide (w/w) were added to the wells. The reaction was started with the addition of 20 μL 18.4 mM TMB dihydrochloride (Sigma Aldrich) in dimethylformamide. Plates were incubated at 37 °C for 10 min, and then the reactions were stopped by the addition of 30 μL of 1.46 M sodium acetate, pH 3.0. Enzyme activity was determined colorimetrically using a plate reader set to measure absorbance at 630 nm and was expressed as mOD/biopsy. Results are shown as mean ± s.e.m of three independent biological replicates.

### 2.19. Statistical Analysis

Statistical analysis of MTT, Quantitative PCR, Calcium Influx, and Mast Cell Degranulation (beta-hexosaminidase) assays were performed using one-way ANOVA post-hoc Tukey test for average comparisons using the GraphPad Prism 6 program (GraphPad Software, San Diego, CA, USA). Statistical significance was established at *p* < 0.1. Statistical analysis of Paw Edema in vivo assay was performed using two-way ANOVA post-hoc Tukey test for average comparisons using the GraphPad Prism 6 program. Statistical significance was established at *p* < 0.1. Statistical analysis of Myeloperoxidase Activity in vivo was performed using Student’s T test for average comparisons using the GraphPad Prism 6 program. Statistical significance was established at *p* < 0.1.

## 3. Results

### 3.1. LiRecTCTP Expression and Purification

**Figure 1:**
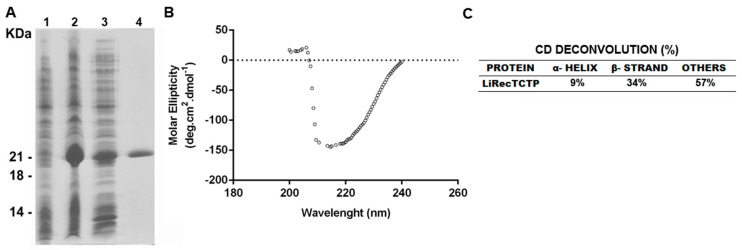
Heterologous expression, purification, and circular dichroism spectroscopy CD analyses of recombinant LiTCTP. (**A**) SDS-PAGE (12.5%) analysis of recombinant LiTCTP toxin expression stained with Coomassie blue dye. Lane 1 show *E. coli* BL21 (DE3) pLysS cells before induction with IPTG. Lane 2 shows *E. coli* BL21(DE3) pLysS after induction for 4h with 0.1 mM isopropyl-d-thiogalactoside (IPTG) (supernatant from cell lysates obtained by freezing and thawing in extraction buffer before incubation with Ni^2+^-NTA beads). Lane 3 depicts the void from Ni^2+^-NTA chromatography. Lane 4 shows eluted recombinant protein from Ni^2+^-NTA beads. Molecular mass markers are shown on the left. (**B**) The UV-CD spectrum was obtained in a Jasco J-810 spectropolarimeter (Jasco Corporation, Tokyo, Japan) by diluting the sample at 0.5 mg/mL in phosphate buffer, pH 7.4 at 20 °C. Graphic representation was plotted using GraphPad Prism 6. (**C**) The deconvolution of data, α-helix and β-sheet percentages of LiRecTCTP structure was calculated using K2D3 tool.

LiRecTCTP expression was performed using the same heterologous system previously described, using *E. coli* and a His-tag [2], but we used an improved protocol of purification. In the former protocol, recombinant toxin was purified in native conditions in a 2-step chromatographic approach: Ni+2-NTA affinity chromatography using, and ion-exchange chromatography using DEAE agarose [2]. Herein, LiRecTCTP was purified under native conditions in just one step of chromatography (Ni^+2^-affinity chromatography). This new protocol resulted in highly purified recombinant toxin and the yield was 16 mg/L of *E. coli* culture (Figure 1A). Purified LiRecTCTP toxin was submitted to circular dichroism to analyze protein folding. Deconvolution results show 43% of defined secondary structures as alpha-helix and beta-sheets (Figure 1B,C). These results are in agreement with previous data obtained for LiRecTCTP [2].

### 3.2. LiRecTCTP Activity on RBL-2H3 Cells

**Figure 2:**
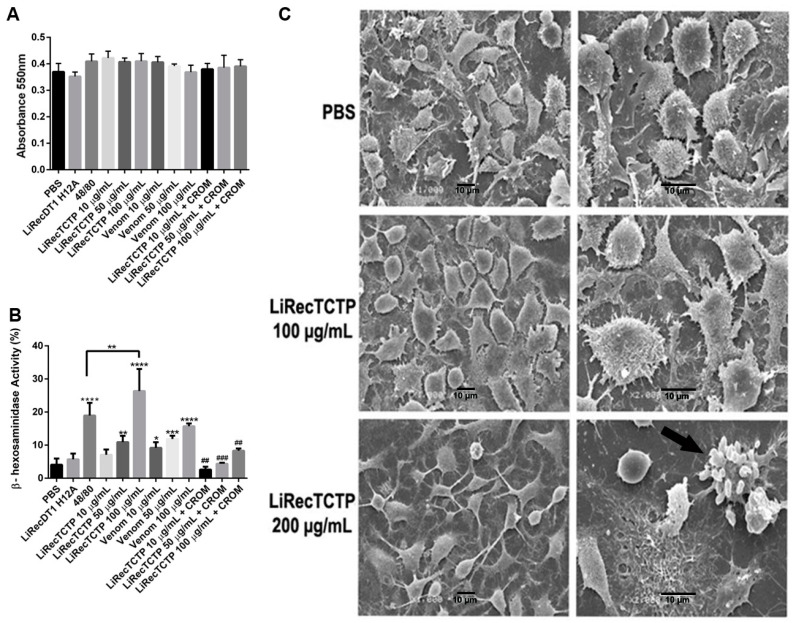
Effects of LiRecTCTP in mast cells viability and degranulation in vitro. RBL-2H3 cells (mast-like cells) were incubated with LiRecTCTP (10, 50, and 100 µg/mL), total venom from *L. intermedia* (10, 50, and 100 µg/mL), compound 48/80 (100 µg/mL) (positive control), LiRecDT1 H12A (100 µg/mL) (negative control) or PBS (negative control). After 2 h viability, morphology and activity of the granular enzyme Beta-hexosaminidase were measured. Inhibition of degranulation was performed using cromolyn (CROM) (20 µM). (**A**) Cell viability was evaluated using MTT assay. The values represent the average of the three independent experiments ± s.d. (performed in pentaplicate). (**B**) Beta-hexosaminidase activity assay. Results are expressed as the percentage of the total beta-hexosaminidase activity present in the cells, after subtracting the activity in the supernatant of unstimulated cells. The values represent the average of the three independent experiments ± s.d. (performed in pentaplicate) (* *p* < 0.1; ** *p* < 0.01; *** *p* < 0.001 and **** p < 0.0001 compared with control; ^##^ *p* < 0.01, ^###^ *p* < 0.001 compared to LiRecTCTP treatment). (**C**) Scanning electron microscopy (SEM) of RBL-2H3 cells after 2 h treatment with LiRecTCTP (100 and 200 µg/mL). Images of each sample represent different fields and magnification. Scale bars indicate 10 μm, magnification 1000× (left) and 2000× (right). Arrow: apoptotic cell.

LiRecTCTP activity on mast cells degranulation was evaluated using RBL-2H3, a mast cell–like cell line, originally a rat basophilic leukemia cell. Initially, a cytotoxic effect of LiRecTCTP (100 μg/mL) on these cells was disregarded by evaluating cells viability (MTT assay) and morphology (SEM) (Figure 2A,C) after 2h-treatment with LiRecTCTP (10, 50, and 100 μg/mL). LiRecDT1H12A, a mutated and almost inactive toxin (only residual levels of activity) produced in the same way (heterologous system and chromatographic purification protocols), was included in the experiment (as negative control), as well as a compound that triggers degranulation (48/80, a positive control). Crude venom activity was also evaluated by MTT assay, and the resulting absorbance did not differ from controls. We did not observe deleterious effects of LiRecTCTP (100 μg/mL) in the viability/activity measured by MTT metabolization in formazan salt (Figure 2A) or in the cellular morphology shown in SEM (Figure 2C). Only 200 μg/mL of LiRecTCTP induced alteration in RBL-2H3 cells morphology (Figure 2C), and this concentration was not used for further functional characterization of LiRecTCTP. RBL-2H3 cells degranulation was measured by the beta-hexosaminidase activity assay (Figure 2B), a widely used test, mainly for research purposes. Beta-hexosaminidase is an acid hydrolase that characterizes lysosomal-derived secretory granules that are released during degranulation; the enzyme activity was measured by using p-nitrophenyl *N*-acetyl-beta-d-glucosamine as colorimetric substrate. The degranulation effect is evident when LiRecTCTP is incubated for 2 h with the cells and this activity was dependent of toxin concentration. It is important to highlight that the mutated toxin (LiRecDT1 H12A), produced following the same methodological procedures as LiRecTCTP, was not able to induce beta-hexosaminidase release, ruling out the possibility of contaminants being involved in the effect (Figure 2B). The activity of beta-hexosaminidase after 50 and 100 μg/mL LiRecTCTP treatments was increased two-fold and three-fold, respectively, when compared to the negative control (LiRecDT1 H12A). As shown, 100 μg/mL of LiRecTCTP had a higher degranulation effect than the positive control 48/80, a well-known polymer which triggers mast cell activation. *L. intermedia* crude venom also activates RBL-2H3 cells degranulation in a concentration-dependent manner. It is important to mention the cromolyn inhibitory effect on LiRecTCTP induced beta-hexosaminidase activity. Cromolyn blocks or reduces the mediators released from mast cells, suggesting a pro-inflammatory mechanism of histamine release induced by the LiRecTCTP toxin.

### 3.3. Effects of LiRecTCTP on the Ca^2+^ Signaling and Cytokines Expression

**Figure 3:**
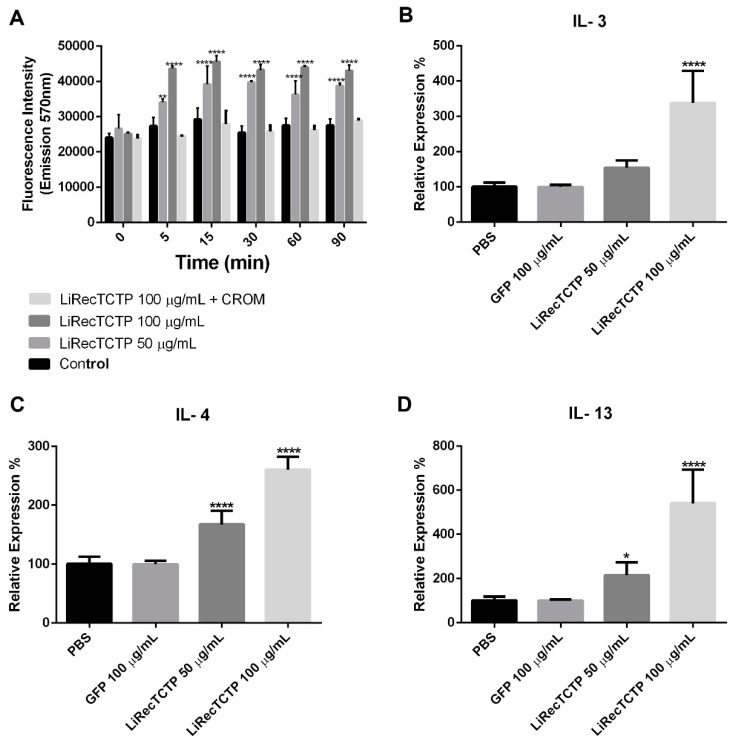
Effect of treatment of RBL-2H3 cells with LiRecTCTP on calcium influx and expression of interleukins. (**A**) RBL-2H3 cells were incubated with LiRecTCTP (50 and 100 µg/mL) in the presence of Fluo-4 AM in buffer containing calcium. The fluorescence of Fluo-4 was measured after 0, 5, 15, 30, 60, and 90 min. As a negative control, cells were incubated without LiRecTCTP. Cromolyn (CROM) (20 µM) inhibitory effect was evaluated in the presence of LiRecTCTP (100 µg/mL). The values represent the average of the three experiments ± s.d. (** *p* < 0.01 and **** *p* < 0.0001). (**B**) Quantitative real-time PCR of IL-3 mRNA levels in RBL-2H3 exposed or not to LiRecTCTP (50 and 100 µg/mL) and GFP (100 µg/mL). (**C**) Quantitative real-time PCR of IL-4 mRNA levels in RBL-2H3 exposed or not to LiRecTCTP (50 and 100 µg/mL). (**D**) Quantitative real-time PCR of IL-13 mRNA levels in RBL-2H3 exposed or not to LiRecTCTP (50 and 100 µg/mL) and GFP (100 µg/mL). For quantification, we used the ΔΔCt method with GAPDH as an endogenous control for each sample (* *p* < 0.1 and **** *p* < 0.0001, compared with controls, PBS and GFP recombinant protein). Data represent mean ± s.d. of three independent experiments.

As changes in the cytosolic Ca^2+^ are central for mast cells activation, we performed an assay to measure the Ca^2+^ influx in RBL-2H3 following a LiRecTCTP treatment (Figure 3A). We could observe a dose-dependent positive effect of LiRecTCTP in the Ca^2+^ influx. Cromolyn abrogated LiRecTCTP effects on Ca^2+^ levels. We also analyzed the cytokine production evoked by LiRecTCTP treatment in RBL-2H3 cells, by measuring the relative mRNA levels for IL-3 (Figure 3B), IL-4 (Figure 3C) and IL-13 (Figure 3D) by means of RT-PCR. LiRecTCTP increased the expression of IL-3, IL-4 and IL-13 in a dose-dependent manner, when compared to the negative controls (PBS and 100 µg/mL of GFP recombinant protein).

### 3.4. LiRecTCTP In Vivo Effects—Vascular Permeability and Edema

**Figure 4:**
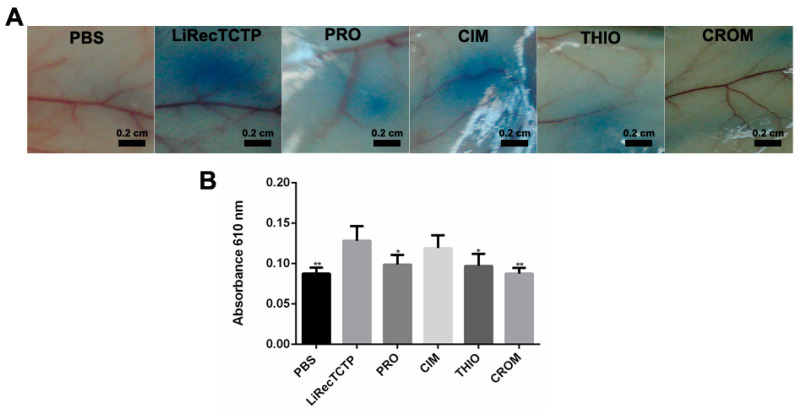
Effect of treatment with the mast cell degranulation inhibitor or histamine receptor antagonists on vascular permeability induced by LiRecTCTP. Mice (*n* = 5) were pre-treated with promethazine (PRO), cimetidine (CIM), thioperamide (THIO), cromolyn (CROM), or PBS (control). Animals received solution of Evans blue dye in PBS intravenously prior to intradermal injection of LiRecTCTP (10 µg) or PBS (control). (**A**) Representative images of mice dorsal skin at the point of sample inoculation. (**B**) Quantitative measurement of dye leakage induced by LiRecTCTP and inhibition with CROM, PRO, CIM, and THIO. Data represent mean ± s.e.m of one representative experiment from three independent biological replicates (* *p* < 0.1 and ** *p* < 0.01).

In order to evaluate the effect of different histamine receptor blockers on the histaminergic response triggered by LiRecTCTP, we performed two animal studies in which vascular permeability and edema formation were assessed. Well-established antihistaminic drugs with different targets were used in these experiments: promethazine (PRO), an H1 receptor antagonist; cimetidine (CIM), an H2 receptor antagonist; thioperamide (THIO), acts on H3 and H4 receptors; and cromolyn (CROM), a mast cell degranulation blocker. Vascular permeability was measured by Evans blue extravasation (Figure 4A) after intradermic inoculation of LiRecTCTP in mice, previously treated with an antihistaminic or not. Quantification was performed after Evans blue elution (Figure 4B). Images from mice skin and amount of dye eluted show that cromolyn was the most effective drug to reduce LiRecTCTP effects on vascular permeability (absorbance of the eluted dye was very similar to the negative control, PBS). Promethazine and thioperamide could inhibit about 30% of the LiRecTCTP effect in increasing vascular permeability. Cimetidine did not alter the increase in vascular leakage of Evans dye caused by LiRecTCTP.

**Figure 5:**
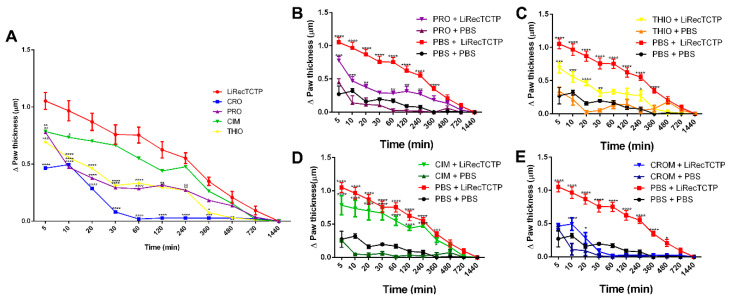
Effect of the treatment with the mast cell degranulation inhibitor or histamine receptor antagonists on edema induced by LiRecTCTP. Mice (*n* = 5) were pre-treated with promethazine (PRO), cimetidine (CIM), thioperamide (THIO), cromolyn (CROM), or PBS (control), and thereafter, animals were injected with LiRecTCTP (10 µg) or PBS (control) into footpads for edema. (**A**) Paw edema observed after injection with LiRecTCTP, in animals previously treated with PBS (LiRecTCTP), mast cell degranulation inhibitor (CRO), or histamine receptor antagonists (PRO, CIM, THIO). (**B**) Paw edema observed after injection with LiRecTCTP or PBS, in animals previously treated with PBS or promethazine (PRO). (**C**) Paw edema observed after injection with LiRecTCTP or PBS, in animals previously treated with PBS or thioperamide (THIO). (**D**) Paw edema observed after injection with LiRecTCTP or PBS, in animals previously treated with PBS or cimetidine (CIM). (**E**) Paw edema observed after injection with LiRecTCTP or PBS, in animals previously treated with PBS or cromolyn (CROM). Values represent the thickness difference between the edema after injection with LiRecTCTP and initial before injections. Each point represents the mean ± s.e.m of five animals from one representative experiment from three independent biological replicates (* *p* < 0.1; ** *p* < 0.01; *** *p* < 0.001 and **** *p* < 0.0001).

We also used mice to evaluate if LiRecTCTP edematogenic effects could be inhibited by the anti-histaminic drugs; the effect of these inhibitors is shown on Figure 5A compared to LiRecTCTP by itself. Cimetidine did not present a significant effect on the paw edema caused by LiRecTCTP; a small inhibition of edema is observed in the first 10 min after toxin administration (Figure 5D). As shown for vascular permeability, promethazine, thioperamide, and cromolyn prevented LiRecTCTP effects on mice paw (Figure 5B,C,E). The time-courses for promethazine and thioperamide had the same profile (Figure 5A). When we compared the effects of these drugs on the paw edema generated by LiRecTCTP, we observed a decrease along the time (5–1440 min). Among tested drugs, cromolyn showed the highest inhibition of LiRecTCTP-induced edema (Figure 5A,E). Figure 5B–E show the anti-histaminic effects and their comparison with respective controls: (i) LiRecTCTP or PBS inoculation in animals previously treated with the drug and (ii) preliminary treatment with PBS and following inoculation of PBS or LiRecTCTP in mice paw. These graphs show there was no significant swelling of the paw after the inoculation of the same volume of PBS and also that the anti-histaminic drugs did not cause unspecific edema. When we compare LiRecTCTP edema curves in the presence of cromolyn and the negative control (PBS), they are very similar (Figure 5E). As observed in permeability assay, cromolyn abrogates LiRecTCTP edematogenic effects.

### 3.5. LiRecTCTP In Vivo Effects—Dermonecrotic Lesion

**Figure 6:**
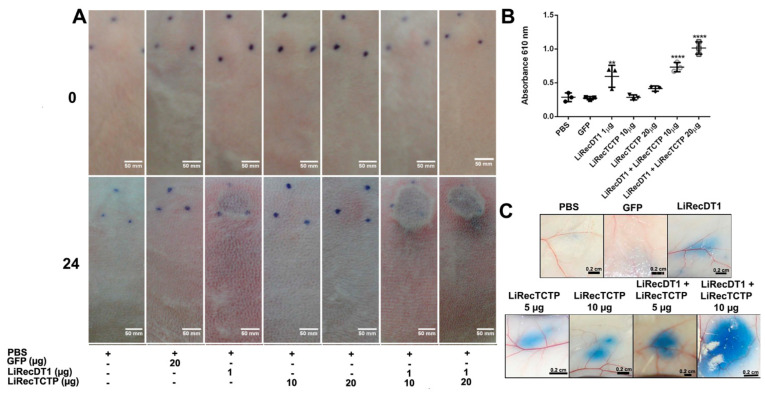
Inflammatory response of combined recombinant toxins (LiRecTCTP and LiRecDT1) in vivo. (**A**) Macroscopic evaluation of rabbit skin exposed to recombinant toxins (LiRecTCTP, LiRecDT1, or combined toxins LiRecTCTP/LiRecDT1). Rabbits were subcutaneously injected with dermonecrotic toxin LiRecDT1 (1 µg, as positive control), LiRecTCTP (10 and 20 µg), LiRecDT1 (1 µg) combined with LiRecTCTP (10 and 20 µg), GFP (20 µg), a recombinant inactive protein (negative control), or PBS (negative control) (+, present; −, absent). Animal skins were photographed just after inoculation (0 h) and 24 h following injection. The same animal received the seven samples for adequate comparison, experiment was repeated twice, using 2 and 4 rabbits respectively. (**B**) Inflammatory reactions induced by toxins and controls were estimated by measurement of myeloperoxidase activity from neutrophils infiltrate at dermis. Values are expressed as mean ± s.e.m of absorbance at 610 nm. Each point represents the average of three replicates from the inoculation site on rabbit skin at the end of experiment (24 h) (** *p* < 0.01 and **** *p* < 0.0001). (**C**) Effect of LiRecTCTP and LiRecDT1 on vascular permeability of skin vessels. Mice were injected intradermally with of LiRecTCTP (5 or 10 µg), LiRecDT1 (1 µg), or recombinant GFP (10 µg) (negative control). PBS was used as a vehicle control. Experiment was performed three times using groups of five mice for each condition. Dye leakage induced by LiRecTCTP combined with LiRecDT1 is higher than the leakage observed with each toxin alone. Scale bar points 0.2 cm.

**Figure 7:**
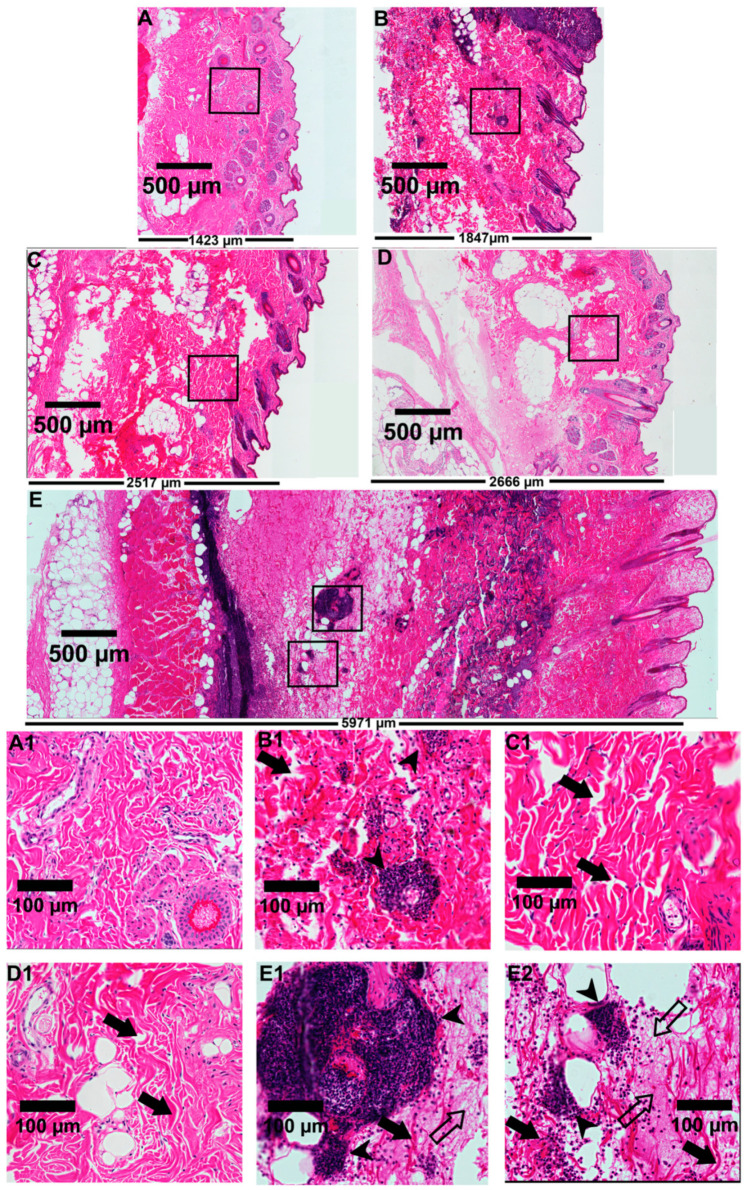
Microscopic evaluation of rabbit skin exposed to recombinant toxins (LiRecTCTP, LiRecDT1, or combined toxins LiRecTCTP/LiRecDT1). Light microscopic analysis of tissue sections was performed on rabbit skin after 24 h of injection. The tissue sections were stained with hematoxylin and eosin. Edema triggered in rabbit skin by (**A**) GFP, (**B**) LiRecDT1 (1 µg), (**C**) LiRecTCTP (10 µg), (**D**) LiRecTCTP (20 µg), and (**E**) the combination of LiRecDT1 (1 µg) and LiRecTCTP (20 µg), as visualized by skin thickness. Skin structures are compared via scanning of images from epidermal (on the right of figure) to muscular tissues (on the left of figure) under the same laboratory conditions (Scale bars indicate 500 µm). The width of the tissue (**E**) points to a deep edema after LiRecTCTP and LiRecDT1 combination when compared to toxins alone (**B**, **D**). Isolated LiRecTCTP (**C**, **D**) induced a higher edema compared to LiRecDT1 alone (**B**) or negative control GFP (**A**), which shows a normal skin histology (**A1**). An intense inflammatory response with the presence of neutrophils and fibrinoid exudates into the dermis is shown when both toxins were administered (**E1**, **E2**) compared to isolated LiRecTCTP (**C1**, **D1**) or LiRecDT1 (**B1**) (Scale bars indicate 100 μm). Closed arrows indicate disorganization of collagen fibers and dermal edema, closed arrowheads indicate a massive inflammatory response with the presence of neutrophils, and open arrows indicate fibrin network deposition. Thickness of the skin tissue (**A**–**E**) is shown in the bottom of each tissue section (µm).

We analyzed the role of LiRecTCTP in the dermonecrotic lesion provoked by *Loxosceles* spiders bite accidents. These necrotic lesions are the hallmark of cutaneous manifestation of *Loxosceles* envenomation. The most studied class of *Loxosceles* toxins is phospholipases-D (also called dermonecrotic toxins) whose biological effects can reproduce the ones observed by crude venom inoculation in rabbit skin [7]. In these experiments, we used LiRecTCTP together with an isoform of *L. intermedia* dermonecrotic toxin, LiRecDT1 [28], to evaluate the synergistic action of these toxins. As a negative control, we used an inactive recombinant protein, GFP, which was produced and purified using the same conditions as LiRecTCTP and LiRecDT1. After the subcutaneous inoculation of toxins in rabbit skin, the site was photographed at the time of inoculation and after 24 h for macroscopic evaluation (Figure 6A). After this time, skin patches were collected and processed for microscopic evaluation by histology analyses (Figure 7). Negative controls show that inoculation (PBS or GFP protein) did not trigger any macroscopic (Figure 6A) or microscopic (Figure 7A,A1) alterations in rabbit skin during our experiment. PBS control did not alter normal skin histology (data not show). Dermonecrotic toxin (LiRecDT1) triggered the hallmark lesion, presenting gravitational spreading (Figure 6A) and a marked inflammatory response, which can be observed in the histological analyses by a great number of neutrophils around blood vessels and diffused to connective tissue surrounding the inoculating site, and development of edema (Figure 7B,B1). As expected, LiRecTCTP alone did not cause a skin lesion, but dose-dependent erythema and edema at inoculation site were observed (Figure 6A). The swelling caused by the toxin is visualized in the histology sections, where the width of the skin is greater in LiRecTCTP samples (Figure 7C,D), when compared to GFP recombinant protein (Figure 7A, negative control) and LiRecDT1 (Figure 7B). Image analysis of histological sections revealed that LiRecDT1 promoted an increase of 41% in the tissue area (from epidermis to adipose) when compared to GFP. LiRecTCTP triggered increased edema of 87% and 91% (compared to GFP), when 10 and 20 μg were used, respectively. The combination of LiRecDT1 and LiRecTCTP 20 μg provoked an increased edema area of 340% when compared to GFP.

Furthermore, dermal edema and disorganization and separation of collagen fibers provide evidence for this swelling (Figure 7C1,D1). The combined use of LiRecDT1 and LiRecTCTP toxins has a synergistic effect on dermonecrosis development—a higher gravitational spreading (Figure 6A) and an increased inflammatory response (Figure 7E) when compared to LiRecDT1 alone (Figure 7B). There are more leucocytes recruited at the lesion site, notably in the papillary dermis, when LiRecTCTP is injected together with the dermonecrotic toxin (Figure 7E), and this number is directly dependent on the dose. Capillary changes with increased permeability, resulting in the passage of plasma into the connective tissue, evidenced by fibrin network formation, is also observed in the presence of both toxins (Figure 7E,E1).

The edema observed in the presence of both toxins was enormous; although macroscopic images do not reveal clearly, this aspect can be verified by respective histology sections, e.g., massive increase in the width of the skin, disorganization of collagen fibers and dermal edema (Figure 7E). In order to quantify the inflammatory response triggered by the toxins in rabbit skin, we evaluate myeloperoxidase activity in the skin patches that were collected (Figure 6B). We can observe an increase of 25% in myeloperoxidase activity when LiRecDT1 was combined with 10 μg LiRecTCTP when compared to LiRecDT1 alone. When 20 μg of LiRecTCTP was used, the increase reaches 65%. LiRecTCTP alone has no significant effect on myeloperoxidase activity; these results were similar to the negative controls (PBS and GFP). We also investigated the synergy of LiRecDT1 and LiRecTCTP in the vascular permeability using mice. Figure 6C shows representative images from Evans Blue assay; dye leakage can be observed after the inoculation of both toxins. This permeability is increased when toxins are administered together, and this was dependent of LiRecTCTP dose. Combination of 10 μg of LiRecTCTP and 1 μg LiRecDT1 administration resulted in a huge and intense blue spot at the inoculation site. There was no relevant extravasation of Evans blue in negative controls (PBS and GFP). A recombinant GFP obtained in the same heterologous system as the *Loxosceles* toxins was used to rule out an unspecific effect due to bacterial contaminants.

## 4. Discussion

Clinical symptoms of envenomation include histamine-related responses; although they are less frequent, there are reports of hypersensitivity or even allergic reactions after spider bites [9,10]. The presence of histamine in the envenomation site of Loxoscelism can cause edema and endothelial changes such as increased vascular permeability and vasodilation, which contribute to the systemic dispersion of venom components and exacerbate the inflammatory response triggered by the bite [7,8]. Inflammatory responses can be related to both mast cells and histamine [6,11]. It has already been shown that *L. intermedia* venom increases vascular permeability and induces vascular relaxation in rats [29] and that these effects are related to the ability of venom to degranulate mast cells and release mediators such as histamine [11].

Different studies that investigated the protective effects of recombinant *Loxosceles* phospholipases-D (PLDs) or even the neutralizing effects of serum produced with these toxins found that the edematogenic activity of *Loxosceles* venoms is particularly difficult to neutralize and raised the possibility that other venom components may be responsible for edema development [30,31]. LiRecTCTP induces an increase in microvascular permeability of skin vessels and is a component of edema formation with an earlier and faster effect when compared to the inflammatory response triggered by whole *L. intermedia* venom in mouse paws [2]. The effects of *L. intermedia* crude venom on RBL-2H3 degranulation (Figure 2C) are related to the histaminergic effects of Loxoscelism. The report of TCTP-related toxins in the *Loxosceles* venoms were previously done by transcriptomic studies using venom gland transcripts [1], by cloning and recombinant expression of LiRecTCTP [2], and recently, TCTP was identified in the proteomic study of the *L. intermedia* venom [32]. Additionally, TCTP was immunodetected in the whole venom of *Loxosceles* species (*L. intermedia*, *L. gaucho*, and *L. laeta*) [3], pointing to conservation and biological relevance. There are described TCTPs from other spiders [33,34], although they are not related to venomous accidents as *Loxosceles* [35].

The toxins purification was a critical procedure for this study: pure and correctly folded LiRecTCTP was obtained (Figure 1). Two other recombinant proteins were used as negative controls to exclude an effect due to a possible prokaryotic contamination from the heterologous expression system: green fluorescent protein (GFP), an innocuous protein, and LiRecDT1 H12A, a mutated isoform derived from LiRecDT1, with drastically reduced activity to residual levels [2,27,36].

In this study of LiRecTCTP’s biological role, it was relevant to rule out its possible cytotoxic effects on RBL-2H3, which could cause release of cellular contents and mimic degranulation in spite of a mechanism dependent of LiRecTCTP action. The morphology of RBL-2H3 cells was not altered by LiRecTCTP, cells submitted to treatment with 100 μg/mL did not differ from control cells, and they remained adhered and spread onto the slide, showing philopodia (Figure 2C). The unreduced capacity to metabolize MTT by cells treated with LiRecTCTP implies a specific effect on degranulation of RBL-2H3 cells (Figure 2A). Beta-hexosaminidase activity after LiRecTCTP treatment of RBL-2H3 cells infers that this toxin can directly trigger degranulation process in these basophil lineage cells (Figure 2B). Cromolyn is known as a “mast cell stabilizer.” It interferes with the release of inflammatory and other chemical mediators from mast cells and either blocks or reduces the amount released [37]. The cromolyn effect as a mast cell stabilizer is dose-dependent.

Human TCTP was already well-characterized as a histaminergic molecule [38,39,40] and suggested as a putative target for therapeutics in asthma and allergy [41,42]. Several reports indicate TCTP involvement in the inflammatory response of parasite-infected individuals [43,44,45,46].

Activation of basophils and mast cells can be monitored through different approaches, including morphological changes, phenotypic changes, and quantification of secreted mediators. IL-4 is produced by basophils in the early response to a stimulus, and IL-3, produced by mast cells, is involved in severe hypersensitivity reactions. Functionally, mast cells, and basophils overlap in their ability to produce several mediators, including histamine and granule proteases. IL-3, IL-4 and IL-13 cytokines act as immunomodulators of other immune cells in the inflammatory and allergic signaling pathway and play pivotal roles in exacerbating the inflammatory responses in vivo [47,48]. Expression of IL-3, IL-4 and IL-13 was induced by LiRecTCTP in RBL-2H3 cells, which indicates that this protein could be involved in the cutaneous inflammatory and histaminic conditions of *Loxosceles* envenomation (Figure 3B). As these cytokines are able to recruit inflammatory immune cells to the bite site, LiTCTP could contribute to the exacerbated inflammatory response by stimulating the production and release of these cytokines. The expression of these cytokines by the RBL-2H3 cells is related to their activation by LiRecTCTP, as indicated by increased Ca^+2^ influx and beta-hexosaminidase activity (Figure 3A,B).

We used different histamine receptors inhibitors in order to evaluate histaminergic effects of LiRecTCTP: H1R to H4R [49]. Alterations in vascular permeability, measured by the leakage of administered Evans blue from vessels (Figure 4), and the edematogenic effect of LiRecTCTP (Figure 5) were performed in mice previously treated or not treated with the anti-histaminic drugs. Cimetidine presented minimum inhibition effect of LiRecTCTP; it could not block toxin-induced permeability and modestly reduced paw edema only in the first 10 min after toxin inoculation. Absence of cimetidine inhibition is explained by the fact that its targets (H2 receptors) are mainly localized in the stomach, brain, and cardiac tissue, but typically not in the skin. Effects of promethazine are related to the blockage of H1R activation by histamine, the previous treatment with this drug resulted in inhibition of LiRecTCTP action on permeability and edema. H1 receptors are expressed by a broad range of cells, including airway and vascular smooth muscle cells, endothelial cells, monocytes, neutrophils, and T and B cells [49]. Thioperamide is a dual H3-H4 receptors antagonist. H3 receptors are irrelevant in the context of our experiments as these histamine receptors are almost exclusively expressed in the nervous system.

On the other hand, histamine H4 receptors are described mainly expressed in cells of the human immune system and influence their cytokine production mediating several effects on chemotaxis [50]. Thioperamide treatment significantly decreased mice response to the histaminergic effects of LiRecTCTP in vascular permeability and paw edema. When the effects of LiRecTCTP on histamine release were blocked by cromolyn, toxin biological effects in animals were almost abrogated: results were similar to the negative controls. These results obtained in animal models point to an in vivo histamine-induced effect by LiRecTCTP, related to mast cell degranulation and histamine effects on H1 and H4 receptors. It is important to mention, in the regard of the pro-inflammatory response triggered by venom and observed in Loxoscelism, that IL-3, IL-4, and histamine can upregulate H1 receptor gene expression. This positive feedback loop could contribute to the exacerbated inflammatory condition seen in dermonecrotic lesions resulted from *Loxosceles* bites. The involvement of H4 receptor in the histaminergic effects of LiRecTCTP should also be highlighted as this receptor is emerging as an essential receptor for the chemoattraction of immunologically relevant cells, contributing to an extended inflammatory cascade.

The investigation of LiRecTCTP participation in dermonecrosis lesion development and clinical features was performed using a well-established in vivo protocol. LiRecDT1 inoculation into rabbit skin, as expected and well-described, caused a characteristic dermonecrosis: a lesion with gravitational spreading, leukocyte infiltration of dermis with the prevalence of neutrophils (PMN), and increased capillary permeability in mice [28,51]. When LiRecTCTP was injected together with the dermonecrotic toxin there was a clear dose-dependent enhancement of all these features at the site of injection: hemorrhage and the gravitational spreading are more intense when compared to LiRecDT1 by itself, and in histopathological analysis, there is an increased number of PMN and more points of fibrin network formation in the connective tissue as a result from an increased microcapillary permeability and disruption of vessel walls (Figure 7). The more prominent cutaneous effect could be explained by the histaminergic effect of LiRecTCTP, which may have contributed to the systemic dispersion of LiRecDT1 toxin. The synergistic effect of both toxins was also revealed by the marked edema and increased dermonecrotic lesions in rabbit skin.

In an envenomation event, LiTCTP is probably acting in edema formation and permeability alterations, corroborating with the huge inflammatory condition of Loxoscelism. LiTCTP could also facilitate the diffusion of other toxins, ultimately promoting venom components spreading from the bite site. Other brown spider toxins that promote extracellular matrix degradation and remodeling, such as metalloproteinases and hyaluronidases, are also implicated in this process [8,52,53]. This combined effect of LiRecDT1 and LiRecTCTP was reproduced in the Evans blue assay. Vascular permeability was highly augmented by the use of both toxins in the inoculation site (Figure 6C). The evaluation of myeloperoxidase activity was crucial to investigate the inflammatory response in mice, as these animals do not develop dermonecrotic lesions (reason still not fully understood) [28,54]. The level of myeloperoxidase (MPO) activity in a sample is directly proportional to the number of neutrophils present, representing the inflammation status [27,55,56]. Results of MPO activity show LiRecTCTP increases the inflammation triggered by LiRecDT1, data that corroborate with its relevant participation in the pro-inflammatory response seen in dermonecrotic lesions (Figure 6B). Concerning the ratio LiRecDT1/LiRecTCTP used in our experiments, transcriptome analysis of *Loxosceles* venoms glands showed that phospholipases-D transcripts such as LiRecDT1 are much more abundant that TCTP transcripts [1]. However, higher concentrations of LiRecDT1 would impair the detection of the synergistic activity between these toxins, inflammatory effects generated only by PLDs would mask the allergenic and inflammatory activities generated by LiTCTP.

Data presented herein confirm previous data that suggested LiRecTCTP histaminergic action by its effect on vascular permeability and edema formation [2]. We suggest that the participation of LiTCTP in the exacerbated inflammatory response is based on LiRecTCTP’s direct effect on mast cells and histamine release. Moreover, LiRecTCTP could be considered for potential applications as biotool, as for instance in basophil activation tests or allergy diagnosis and in vitro testing [8,57].

Altogether, the effects observed for LiRecTCTP, resulting in increased inflammatory response, capillary permeability and edema, as well as acting synergistically with dermonecrotic toxin LiRecDT1, unveil LiTCTP’s role as an additional spreading factor present in the venom of *Loxosceles* spiders. Along with the classic spreading agents described in the venom [13,53], LiTCTP through its histaminergic mechanisms can contribute to the spread of other toxins from the bite site, accentuating the local and even systemic post-envenoming condition.
