# Correction: Boia-Ferreira et al. TCTP from *Loxosceles Intermedia* (Brown Spider) Venom Contributes to the Allergic and Inflammatory Response of Cutaneous Loxoscelism. *Cells* 2019, *8*, 1489

**DOI:** 10.3390/cells13131111

**Published:** 2024-06-27

**Authors:** Marianna Boia-Ferreira, Kamila G. Moreno, Alana B. C. Basílio, Lucas P. da Silva, Larissa Vuitika, Bruna Soley, Ana Carolina M. Wille, Lucélia Donatti, Katia C. Barbaro, Olga M. Chaim, Luiza Helena Gremski, Silvio S. Veiga, Andrea Senff-Ribeiro

**Affiliations:** 1 Department of Cell Biology, Federal University of Paraná, Curitiba 81531-980, PR, Brazil; marianna.boia@gmail.com (M.B.-F.); kamilamoreno7@gmail.com (K.G.M.); alana_abcb@hotmail.com (A.B.C.B.); spedrosa.lucas@gmail.com (L.P.d.S.); larissavuitika2@gmail.com (L.V.); donatti@ufpr.br (L.D.); or ochaim@ucsd.edu (O.M.C.); luiza_hg@yahoo.com.br (L.H.G.); veigass@ufpr.br (S.S.V.); 2 Department of Pharmacology, Federal University of Paraná, Curitiba 81531-980, PR, Brazil; brunasoley@gmail.com; 3 Department of Structural and Molecular Biology, State University of Ponta Grossa, Ponta Grossa 84030-900, PR, Brazil; anacarolina.wille@yahoo.com.br; 4 Laboratory of Immunopathology, Butantan Institute, São Paulo 05503-900, SP, Brazil; katiacbarbaro@hotmail.com; 5 Department of Pharmacology, School of Medicine, University of California San Diego, La Jolla, CA 92093, USA

In the original publication [1], there was a mistake in Figure 6A as published. The image of the PBS (control)-injected rabbit at a time of 0 h was unintentionally repeated for the conditions DT1 and TCTP 20 mg/kg. The corrected Figure 6 appears below. The authors state that the scientific conclusions are unaffected. This correction was approved by the Academic Editor. The original publication has also been updated.

## Figures and Tables

**Figure 6:**
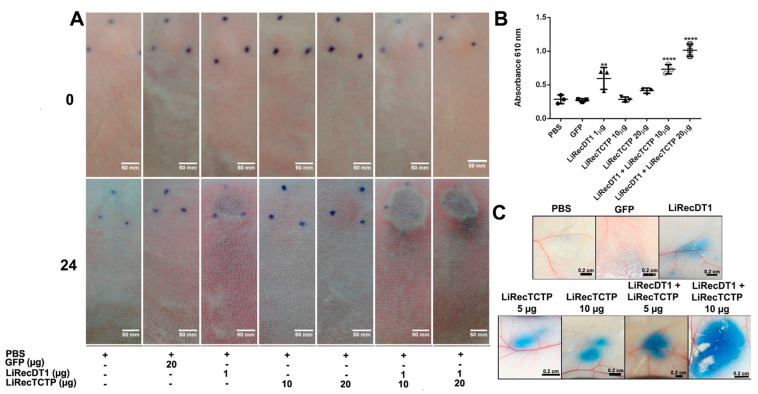
Inflammatory response of combined recombinant toxins (LiRecTCTP and LiRecDT1) in vivo. (**A**) Macroscopic evaluation of rabbit skin exposed to recombinant toxins (LiRecTCTP, LiRecDT1, or combined toxins LiRecTCTP/LiRecDT1). Rabbits were subcutaneously injected with dermonecrotic toxin LiRecDT1 (1 µg, as positive control), LiRecTCTP (10 and 20 µg), LiRecDT1 (1 µg) combined with LiRecTCTP (10 and 20 µg), GFP (20 µg), a recombinant inactive protein (negative control), or PBS (negative control) (+, present; −, absent). Animal skins were photographed just after inoculation (0 h) and 24 h following injection. The same animal received the seven samples for adequate comparison, experiment was repeated twice, using 2 and 4 rabbits respectively. (**B**) Inflammatory reactions induced by toxins and controls were estimated by measurement of myeloperoxidase activity from neutrophils infiltrate at dermis. Values are expressed as mean ± s.e.m of absorbance at 610 nm. Each point represents the average of three replicates from the inoculation site on rabbit skin at the end of experiment (24 h) (** *p* < 0.01 and **** *p* < 0.0001). (**C**) Effect of LiRecTCTP and LiRecDT1 on vascular permeability of skin vessels. Mice were injected intradermally with of LiRecTCTP (5 or 10 µg), LiRecDT1 (1 µg), or recombinant GFP (10 µg) (negative control). PBS was used as a vehicle control. Experiment was performed three times using groups of five mice for each condition. Dye leakage induced by LiRecTCTP combined with LiRecDT1 is higher than the leakage observed with each toxin alone. Scale bar points 0.2 cm.
